# Large-scale analysis of structural, sequence and thermodynamic characteristics of A-to-I RNA editing sites in human Alu repeats

**DOI:** 10.1186/1471-2164-11-453

**Published:** 2010-07-28

**Authors:** Yoav Kleinberger, Eli Eisenberg

**Affiliations:** 1 Raymond and Beverly Sackler School of Physics and Astronomy, Tel Aviv University, Tel Aviv, Israel

## Abstract

**Background:**

Alu repeats in the human transcriptome undergo massive adenosine to inosine RNA editing. This process is selective, as editing efficiency varies greatly among different adenosines. Several studies have identified weak sequence motifs characterizing the editing sites, but these alone do not account for the large diversity observed.

**Results:**

Here we build a dataset of 29,971 editing sites and use it to characterize editing preferences. We focus on structural aspects, studying the double-stranded RNA structure of the Alu repeats, and show the editing frequency of a given site to depend strongly on the micro-structure it resides in. Surprisingly, we find that interior loops, and especially the nucleotides at their edges, are more likely to be edited than helices. In addition, the sequence motifs characterizing editing sites vary with the micro-structure. Finally, we show that thermodynamic stability of the site is important for its editing.

**Conclusions:**

Analysis of a large dataset of editing events reveals more information on sequence and structural motifs characterizing the A-to-I editing process

## Background

RNA Editing is a post-transcriptional modification of mRNA [1-4], which may result in the synthesis of proteins that are not directly encoded in the genome. There are two major types of RNA Editing in mammals, both of which occur via deamination of a base, either cytidine (which is turned into uridine) or adenosine (which turns into inosine). Inosine is read by the ribosome (and sequencers) as guanosine, and thus A → I modifications at the mRNA level translate into an A → G changes at the genetic code level. In this work we focus exclusively on A-to-I RNA Editing, which is catalyzed by enzymes from the ADAR (Adenosine Deaminases that Act on RNA) family. ADARs are double-stranded RNA (dsRNA) binding proteins, and thus dsRNA is a prerequisite for A-to-I editing [1,2].

RNA Editing is a fine-tuning mechanism, capable of changing only a few nucleotides. Both edited and unedited variants of the same transcript may be present in the cell. A-to-I editing is known to be vital in vertebrates, and important for normal life in invertebrates. In *Drosophila*, knocking out ADAR activity causes the flies to exhibit defects in locomotion and mating and to suffer tremors [5]. ADAR knockout *C. elegans *worms exhibit chemotaxis defects [6]. In mice, knocking out ADAR1 causes embryonic death and defects in erythropoiesis [7,8]. ADAR2 -/- mice die shortly after birth and are increasingly seizure prone after postnatal day 12 [9]. The lethal phenotype is accounted for by a single editing site resulting in a single amino acid substitution in the gluR-B gene.

In addition, alteration of A → I editing has been ascribed to several pathological conditions [10], mainly to neuro-psychiatric conditions such as amyotrophic lateral sclerosis (ALS) [11], epilepsy [9,12], major depression disorder [13-15], and glioblastoma multiforme [16]. Reduced A-to-I editing levels have been linked to cancer in various tissues, most strongly to brain tumors. A correlation between the reduction of ADAR3 and the tumor aggressiveness was observed, and overexpression of ADAR1 and ADAR2 resulted in decreased proliferation rate of the glioblastoma multiforme cell-lines [17].

Isolating inosine-containing transcripts from *C. elegans *and human brain, it has been noticed that most A-to-I editing occurs in non coding regions [18]. Genome-wide bioinformatic searches for A-to-I editing sites have enabled the identification of abundant A-to-I editing in the transcriptome of several vertebrates [19-24]. It was found that editing occurs mainly within repetitive elements. These repetitive elements are likely to base-pair with a neighboring similar element and form the dsRNA structure which is the target of the ADAR enzymes. In particular, virtually all A-to-I editing events in human occur specifically within *Alu *repeats.

The *Alus *are a particular set of primate-specific retrotransposons, approximately 280 nucleotides in length. The *Alus *are the most abundant of all transposable elements in primates, making up more than 10% of the human genome, with some 1.1 million copies. Recent studies [21,23] have demonstrated that the frequency of A-to-I editing in human is much higher than in mouse, rat, chicken and fly. This has to do with the abundance and low diversity of the *Alu *elements as compared to similar elements in other genomes [24]: since *Alu *is so common in the human genome, there is a high probability that an *Alu *and a counterpart, oppositely oriented *Alu*, exist nearby and are transcribed together. When the RNA transcript folds, these two *Alus *form a helix, thus becoming a target for the dsRNA binding ADARs.

The physiological significance of A-to-I editing within non-coding repetitive elements is still elusive. Several possible mechanisms have been suggested through which editing of a non-coding repetitive element might affect the fate of a transcript: editing may result in insertion or elimination of a splice site, and may theoretically lead to the alteration of transcriptional start and stop codons [25]. Hyperedited inosine-containing RNAs might be cleaved at specific sites [26-29]. In addition, inosine containing mRNAs were also shown to be retained in the nucleus, suggesting an additional regulatory role for A-to-I editing [30,31]. However, the validity and scope of this last mechanism has been debated recently [32,33]. Finally, while the molecular significance is yet unclear, editing within *Alu *repeats was shown to be altered in cancerous tissues [17].

A-to-I editing is characterized by a puzzling specificity and selectivity in the adenosines which are edited. In some substrates, e.g. the AMPA receptor gluR-B subunit in mice [34] and the E1 sites within an *Alu *repeat in the NARF gene [25], RNA Editing is extremely efficient, editing 100% of transcripts at a specific adenosine. In others, such as most of the sites in *Alu *repeats, a seemingly random editing pattern is observed, where many adenosines are targeted, with varying editing efficiency. However, careful analysis reveals that editing in *Alu *repeats is also highly reproducible: the variability among healthy individuals in editing level at a given site within a specific *Alu *repeat is much lower than the site-to-site differences. Sequence preferences for ADARs have been previously documented. C and T are overrepresented at the nucleotide 5' to the editing site, while G is underrepresented. At the nucleotide 3' to the site, G is significantly overrepresented [19,35-39]. These motifs are too weak, however, to fully characterize A-to-I editing. Therefore, the question still stands: what controls the editing level at each given site? ADARs bind to the RNA via double-stranded RNA binding motifs. Thus, dsRNA is a necessity for A-to-I editing. Indeed, it has been shown for the highly selective R/G editing site within the hairpin of the glutamate receptor subunits mRNAs, that the identities of bases in the helical region are evolutionarily conserved, while the bases in the nonhelical part of the hairpin covary so as to maintain their non-helical structure [40]. This distinctive feature demonstrates the importance of the secondary structure to the phenomenon of RNA Editing.

The internal structure of the dsRNA is expected to control the editing efficiency [41]. For example, it has been shown experimentally that internal loops may effectively be equivalent to helix termini in terms of editing efficiency [42]. Thus, internal loops along dsRNA, if large enough, may act as delimiters separating a large dsRNA into many small helices. Since ADARs deaminate fewer A's in shorter helices, their existence (along with the sequence preferences of the ADARs) might be a means to increase the specificity of editing. It is thus plausible that more features of the secondary structure of an RNA molecule play an important role in determining the specificity of adenosine deamination of an ADAR substrate.

In this paper we will characterize the properties of A-to-I editing sites in terms of their secondary structure properties, their sequence properties, and their thermodynamic properties. We describe the building of a database of MFOLD[43] foldings used to query these properties, and then display and discuss the results of those queries.

## Results and Discussion

### Structural Analysis

**Figure 1:**
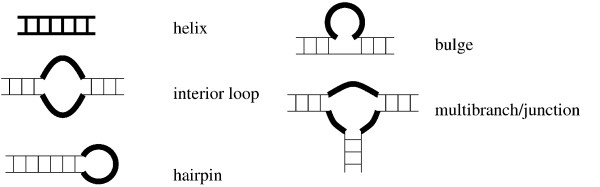
**Secondary structure substructures**. Bold lines indicate those nucleotides formally included in a given substructure.

We first look at the editing frequency for each substructure type (see Table 1 and fig. 1). We compare a "test set" of A-to-I Editing sites, which we denote by *E*^1^, and a control set of sites not known to be edited, denoted by *E*^0^. The *E*^1 ^and *E*^0 ^sets are defined with precision in the Methods section. Interestingly, while the existence of a helix is well known to be a prerequisite for editing, the overall frequency of *E*^1 ^is actually more than two fold lower in helices (0.044) than in interior loops (0.091). As the overwhelming majority of *E*^1 ^sites reside in helices and interior loops, we focus henceforth on these two substructures only. For clarity, we emphasize here that by "interior loop" we mean only the unpaired nucleotides that form the loop's constituent strands.

**Table 1:** Editing frequency and average structure size for the various substructures.

substructure	count	***E*^1 ^freq**.	average size(*E*^0^)	average size(*E*^1^)
bulge	14941	0.031	5.74 ± 0.11	3.44 ± 0.11
hairpin	25318	0.032	8.98 ± 0.09	7.82 ± 0.37
helix	395255	0.044	12.47 ± 0.03	14.08 ± 0.16
interior	107112	0.091	5.61 ± 0.03	4.00 ± 0.05
junction	74788	0.024	23.56 ± 0.10	17.20 ± 0.49
strand	2763	0.020	13.38 ± 0.45	9.20 ± 1.74

*size *is the number of nucleotides in the structure for bulge, hairpin, interior, junction and strand, and the length of the helix for helices. The table lists average sizes with 95% confidence intervals.

"strand" here means an RNA strand which is not part of a loop (stand-alone strand).

**Figure 2:**
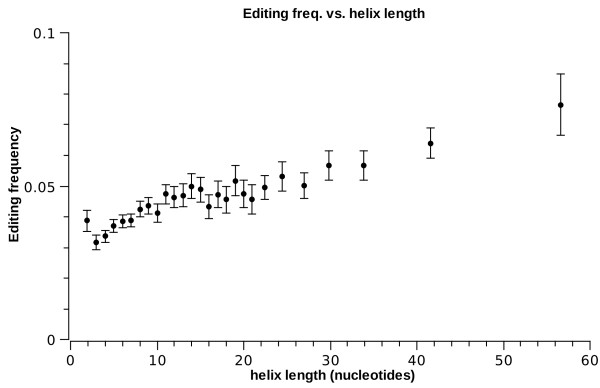
***E*^1 ^frequency vs. helix length**.

**Figure 3:**
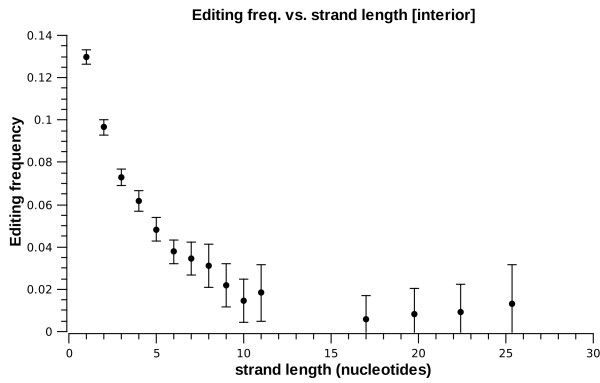
***E*^1 ^frequency vs. interior-loop strand length**.

**Figure 4:**
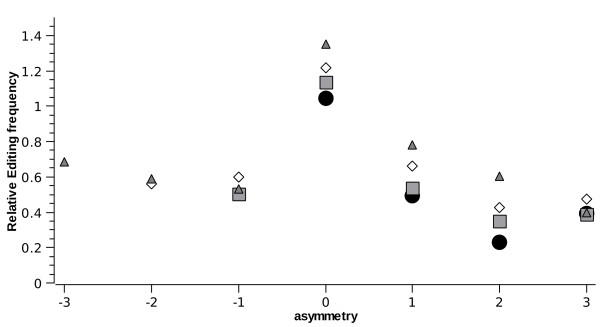
***E*^1 ^frequency decreases with asymmetry**. Editing frequency is presented for sites within interior-loop strands of lengths 1 (circles), 2 (squares), 3 (diamonds), 4 (triangles), as a function of the asymmetry of the loop. Asymmetry is defined as the difference between the length of the strand opposing the editing site and the edited strand length. Frequencies are normalized by the averaged editing frequency for sites having same strand length, regardless of opposite strand length.

Table 1 also suggests length dependence. The editing prevalence as a function of length is given in figs. 2 and 3 (henceforth, error bars represent 95% confidence intervals. Also, some graphs of integer-valued variables have non-integer entries due to data binning). Clearly, longer helices are more likely to be edited, while longer strands of interior loops are less likely to be edited. In addition, the length of the opposite strand (the one the editing site does not reside in) also affects the editing frequency in an interior loop: as shown in fig. 4, symmetric loops are more likely to be edited.

**Figure 5:**
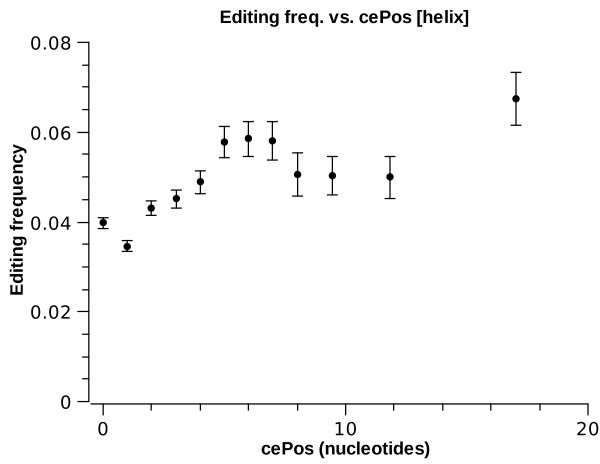
***E*^1 ^frequency vs. ****cePos**** for helices**.

**Figure 6:**
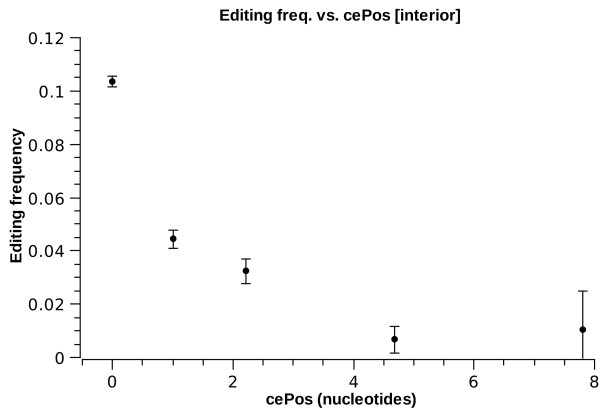
***E*^1 ^frequency vs. ****cePos**** for interior loops**.

Furthermore, we study the effect of the location of the specific nucleotide within its respective substructure. We define cePos as the distance of the site (in nucleotides) from the *closest edge *of the substructure it is in (cePos = 0 means the very edge of a substructure). Figs. 5 and 6 present the frequency of *E*^1 ^sites as a function of cePos. For helices, one observes a general trend of enhancement of editing as a site lies deeper in the helix. For interior loops, however, there is dramatic depletion of *E*^1 ^for cePos > 0. In fact, it should be noted that 91% of edited sites in interior loops lie at the very edge of the loop, i.e. cePos = 0. Most of these are in fact a single mismatch within an almost perfect helix (i.e., opposite strand length is also one nucleotide). Such mismatches were already implicated as preferred targets of ADARs, as previous in-vitro data as well as bioinformatic work indicate that AC mismatches are more favorable substrates than A-T pairs [19,44]. However, it is worthwhile noticing that our analysis shows this trend to persist even for longer interior loops: interior loop strands of length up to five nucleotides are more likely to be edited than the average site in a helix (see fig. 3).

For these cePos = 0 sites, there is a significant (*p *< 2.2e-16) effect to the direction of the nearest neighboring helix: A-to-I editing frequency is 0.068 for sites with a helix only in the upstream site, 0.094 for sites with a helix only in the downstream site, and 0.13 for sites with helices on both sides.

The above results hold when controlling for the total length of the substructure: we compared *E*^1 ^and *E*^0 ^sites for helices of a given length, and for loops of a given size. The resulting trend was the same: for *E*^1 ^sites in helices cePos is larger than for *E*^0 ^sites, whereas in interior loops the connection is reversed. Other location variables tested, such as the position relative to the middle of the substructure, or relative to the 5' end, did not result in noticeable results.

### Sequence Analysis

We start with the nucleotide opposite of the editing site. For helices, it is clear what this means: the "opposite" nucleotide of a site is the nucleotide that pairs with that site (and is therefore always T). We expand this idea, however, to sites at the edges of interior loops (i.e., having cePos = 0): for these sites on the most 5' (3') nucleotide of the loop-strand, the opposite nucleotide is the most 3' (5') site of the other strand in the loop. If the site is the only one on its strand, and the opposite strand has more than one nucleotide, the opposite nucleotide is undefined. We shall refer to the opposite nucleotide as opNuc for short.

There is a very strong enrichment for sites with C on the opposite site: we looked at the frequency of *E*^1 ^for sites with a given opNuc, and obtained a frequency of 18.5% for C, whereas for A the frequency was 5.1% and for G, 3.7%. This is consistent with (but more pronounced than) the data presented in [19-22,37,44].

**Figure 7:**
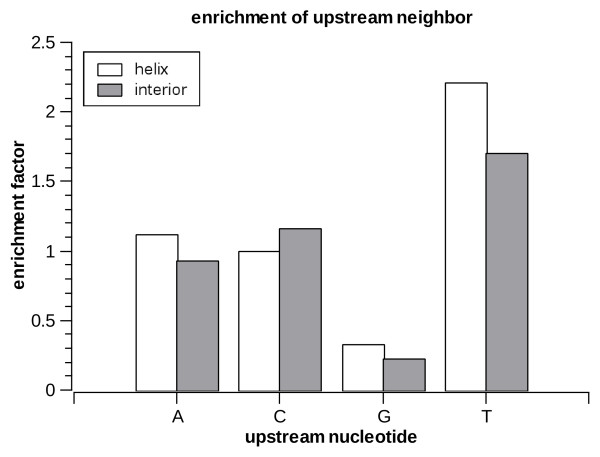
**Enrichment factors for upstream nucleotide in helices and interior loops**.

**Figure 8:**
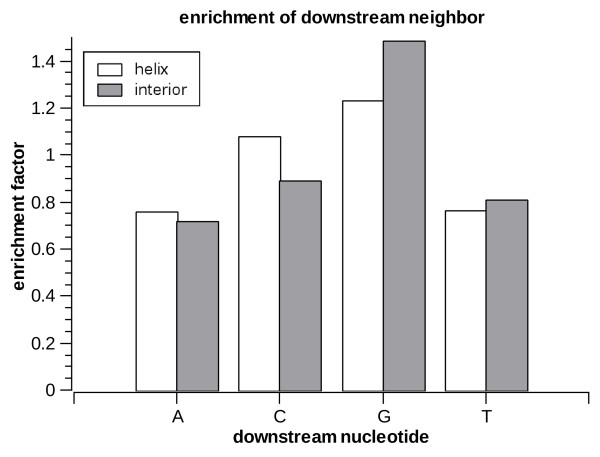
**Enrichment factors for downstream nucleotide in helices and interior loops**.

**Figure 9:**
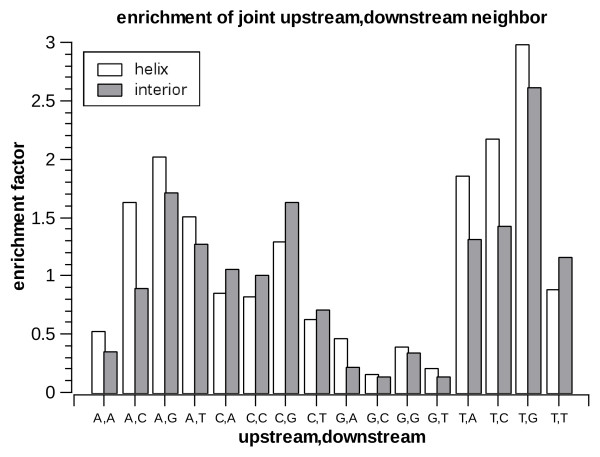
**Enrichment factors for joint upstream, downstream nucleotides in helices and interior loops**.

Next we look at the statistics of the nucleotides upstream and downstream of the A-to-I editing sites. In order to avoid biases due to the underlying nucleotide statistics in *Alu *repeats we do not look at the raw distribution of nucleotides but rather at the enrichment factor, i.e. how much is the editing frequency increased (compared to the average within the respective substructure) when the neighboring site is any specific nucleotide. The enrichment factors are presented in figs 7, 8 and 9 for the two immediate neighbor nucleotides separately, as well as for the *joint *variable composed of both upstream and downstream neighbors. Overall, the profiles found are similar to those seen in previous large-scale studies of editing [19-22,24,45]: T is most preferred upstream and is not preferred downstream, while G is most preferred downstream and least preferred upstream (in both helices and loops). However, we do find a significant (*p *< 1.1e-16 for all comparisons) difference between the profiles for helices and loops. For example, the preference for an upstream T is stronger in helices, whereas the preference for a downstream G is stronger in interior loops.

We also calculated the enrichment factors for the joint variable composed of the site's upstream neighbor, downstream neighbor, and opNuc. The results are displayed in Table 2.

**Table 2:** Frequency of *E*^1 ^and enrichment factors for the joint distribution of upstream neighbor, downstream neighbor and opNuc.

up1,dn1:opNuc	***E*^1 ^freq**.	enrichment factor	# of sites
G,T:A	0.005	0.054	407
G,T:G	0.006	0.066	501
G,C:A	0.006	0.069	160
G,C:G	0.007	0.081	273
G,G:G	0.007	0.081	545
A,C:G	0.014	0.156	213
G,A:G	0.016	0.174	190
C,C:G	0.019	0.207	319
C,T:G	0.019	0.213	362
G,C:C	0.021	0.226	1268
C,T:A	0.022	0.240	412
G,G:A	0.023	0.256	1078
C,A:A	0.024	0.265	334
A,A:A	0.026	0.288	422
G,A:C	0.026	0.292	981
A,A:G	0.030	0.335	461
A,T:G	0.033	0.367	210
T,T:A	0.033	0.368	449
G,T:C	0.034	0.370	1016
T,T:G	0.035	0.383	202
C,C:A	0.037	0.408	784
T,C:G	0.041	0.454	170
G,A:A	0.042	0.468	118
C,A:G	0.044	0.481	528
C,G:G	0.046	0.506	567
A,T:A	0.055	0.608	236
A,C:A	0.059	0.651	339
G,G:C	0.067	0.737	1482
C,G:A	0.068	0.753	966
T,C:A	0.072	0.792	209
A,A:C	0.094	1.035	1226
T,A:G	0.096	1.063	197
T,A:A	0.104	1.149	96
T,G:G	0.110	1.209	228
C,T:C	0.129	1.421	1203
A,G:G	0.144	1.587	278
T,G:A	0.148	1.629	474
C,C:C	0.163	1.801	2321
A,G:A	0.166	1.825	278
C,A:C	0.174	1.923	1515
A,C:C	0.193	2.132	1314
A,T:C	0.248	2.740	1610
C,G:C	0.257	2.839	2797
A,G:C	0.268	2.952	1364
T,T:C	0.269	2.967	788
T,C:C	0.271	2.994	818
T,A:C	0.284	3.138	689
T,G:C	0.374	4.123	1589

In addition, we searched for enrichment in the extended neighborhood of the editing sites, looking at 30 neighboring nucleotides at both sides of the site (up*N *refers to the nucleotide *N *sites upstream to the editing site, and dn*N *refers to the nucleotide *N *sites downstream to the editing site). Almost all neighbors show a significantly different nucleotide distribution around edited sites, see Tables 3 (helices) and 4 (interior loops). The most significant differences (largest χ^2 ^scores) are observed for neighbors up1, up2, up7 and dn18 in helices and up1, dn1, up2 and up3 in interior loops. We note that while almost all 60 neighbors tested show statistically significant difference, it is hard to tell whether these differences are due to ADARs preference or rather stem from editing hot spots within the Alu. We also present the enrichment factors for seven positions surrounding the editing sites which were reported to show preferences to specific nucleotides when surrounding ADAR2 editing sites [41]. As seen in Table 5, the patterns observed here for *Alu *editing are somewhat different: for example, locations dn10 and dn13 seem to favor G in contrast to the opposite trend reported in [41] for ADAR2 sites. The differences might be due to the much larger sample we study here. Additionally, it is also possible that editing sites in the coding region, mostly having a functional role, have different characteristics than the ones in Alu repeats. However, these differences could also result from differences between the profiles of ADAR1 and ADAR2. While the sample of editing sites studied in [41] is biased towards ADAR2 targets, the sample studied here, coming from a wide range of tissues, represents a different mix of the two enzymes, with larger weight of ADAR1. Moreover, the different splice-variants of the ADARs possibly have varying editing efficiencies and site preferences. The mix of these variants occurring in our in-vivo sample, could also lead to slight variations in the preferences observed as compared to results of in-vitro studies.

**Table 3:** Comparison of nucleotide distribution for sites in the vicinity of *E*^1 ^and *E*^0 ^sites in helices.

Neighbor	χ^2^								
-30	160.26	0.2762	0.2359	0.2812	0.2067	0.2378	0.2511	0.3092	0.2019
-29	194.86	0.2602	0.3016	0.2323	0.2059	0.2252	0.3003	0.2737	0.2009
-28	149.27	0.1804	0.2532	0.2360	0.3304	0.1831	0.2848	0.2414	0.2907
-27	51.33	0.1941	0.2644	0.2579	0.2836	0.2148	0.2563	0.2603	0.2686
-26	127.84	0.2433	0.2696	0.3097	0.1775	0.2475	0.2602	0.2831	0.2093
-25	146.16	0.2481	0.3034	0.2765	0.1720	0.2677	0.2643	0.2989	0.1691
-24	559.56	0.2381	0.2516	0.2442	0.2661	0.2908	0.2206	0.2798	0.2088
-23	116.79	0.2108	0.2729	0.3005	0.2158	0.2385	0.2510	0.2818	0.2288
-22	274.04	0.2386	0.2671	0.3280	0.1663	0.2588	0.2788	0.2719	0.1905
-21	361.42	0.1842	0.2367	0.3055	0.2736	0.2084	0.2608	0.2425	0.2884
-20	488.07	0.1840	0.2995	0.2788	0.2376	0.2232	0.2733	0.2232	0.2803
-19	447.66	0.2161	0.3301	0.2585	0.1954	0.2474	0.2614	0.2984	0.1928
-18	41.33	0.2064	0.2542	0.2599	0.2795	0.2186	0.2478	0.2716	0.2620
-17	133.29	0.1968	0.3006	0.2446	0.2580	0.2301	0.2720	0.2476	0.2503
-16	487.44	0.1912	0.2881	0.3059	0.2148	0.2526	0.2696	0.2497	0.2281
-15	54.11	0.1885	0.2952	0.2438	0.2725	0.2058	0.2809	0.2307	0.2826
-14	272.67	0.2501	0.2100	0.2827	0.2572	0.2447	0.2641	0.2500	0.2412
-13	147.24	0.2836	0.2594	0.2857	0.1713	0.2783	0.2888	0.2499	0.1830
-12	520.86	0.2269	0.2540	0.2710	0.2482	0.2255	0.3163	0.2074	0.2508
-11	451.16	0.1812	0.2835	0.2583	0.2770	0.2520	0.2614	0.2426	0.2440
-10	42.99	0.2679	0.2891	0.1903	0.2526	0.2681	0.2685	0.2033	0.2601
-9	114.34	0.2574	0.2715	0.2630	0.2081	0.2693	0.2449	0.2512	0.2345
-8	422.79	0.2130	0.2643	0.2539	0.2687	0.2685	0.2106	0.2619	0.2590
-7	1058.19	0.1511	0.3001	0.2043	0.3445	0.2331	0.2328	0.2429	0.2911
-6	174.05	0.2393	0.2353	0.3030	0.2224	0.2713	0.2333	0.2623	0.2332
-5	471.67	0.2435	0.2610	0.3035	0.1919	0.2890	0.2057	0.2803	0.2249
-4	262.05	0.2098	0.2465	0.2582	0.2856	0.2513	0.2657	0.2285	0.2544
-3	66.91	0.2704	0.2366	0.2404	0.2526	0.2841	0.2230	0.2582	0.2347
-2	1440.18	0.1643	0.2998	0.2271	0.3089	0.2895	0.2510	0.2245	0.2350
-1	5907.22	0.2886	0.3223	0.0918	0.2973	0.2576	0.3229	0.2921	0.1274
1	815.29	0.1975	0.2231	0.4573	0.1221	0.2637	0.2059	0.3682	0.1622
2	514.01	0.2274	0.3116	0.2008	0.2601	0.2918	0.2480	0.2102	0.2500
3	581.33	0.2055	0.3484	0.1837	0.2623	0.2872	0.2998	0.1810	0.2320
4	790.83	0.2177	0.2703	0.2854	0.2266	0.2691	0.2637	0.2032	0.2640
5	432.69	0.2395	0.2605	0.2970	0.2029	0.3035	0.2132	0.3007	0.1826
6	270.68	0.2612	0.1544	0.3715	0.2129	0.2879	0.1804	0.3148	0.2169
7	404.67	0.2103	0.3120	0.2573	0.2203	0.2508	0.2480	0.2791	0.2222
8	375.77	0.2373	0.2568	0.3026	0.2034	0.2902	0.2094	0.2820	0.2184
9	91.53	0.2882	0.2383	0.2245	0.2490	0.2887	0.2560	0.2354	0.2199
10	211.51	0.2553	0.2543	0.2786	0.2118	0.2897	0.2268	0.2496	0.2338
11	697.41	0.1906	0.3458	0.1663	0.2973	0.2691	0.3119	0.1806	0.2384
12	380.76	0.2003	0.2729	0.1865	0.3403	0.2549	0.2725	0.1893	0.2834
13	289.45	0.2619	0.2568	0.2916	0.1897	0.3117	0.2589	0.2434	0.1860
14	855.86	0.2283	0.2950	0.3352	0.1415	0.2767	0.2746	0.2526	0.1961
15	377.73	0.1799	0.2594	0.3243	0.2364	0.2367	0.2333	0.2833	0.2467
16	402.31	0.2394	0.2383	0.3174	0.2049	0.2802	0.2125	0.2660	0.2413
17	155.61	0.2600	0.2783	0.2547	0.2069	0.2540	0.2456	0.2593	0.2412
18	1056.40	0.1928	0.2101	0.3949	0.2022	0.2533	0.2331	0.2827	0.2308
19	951.47	0.2007	0.2938	0.3211	0.1843	0.2826	0.2260	0.2729	0.2184
20	243.36	0.2246	0.3339	0.2596	0.1820	0.2643	0.2864	0.2542	0.1952
21	571.53	0.1781	0.2857	0.2551	0.2811	0.2571	0.2505	0.2461	0.2463
22	725.43	0.2652	0.2713	0.3065	0.1570	0.2978	0.2464	0.2368	0.2190
23	28.63	0.2621	0.2540	0.3017	0.1822	0.2754	0.2568	0.2844	0.1833
24	499.38	0.2649	0.2733	0.2983	0.1636	0.3206	0.2269	0.2580	0.1946
25	141.56	0.2707	0.2457	0.2939	0.1897	0.3110	0.2284	0.2702	0.1905
26	543.54	0.3520	0.2268	0.2674	0.1538	0.2898	0.2239	0.2670	0.2193
27	220.85	0.2635	0.2715	0.2789	0.1861	0.2514	0.2782	0.2433	0.2271
28	568.72	0.1849	0.2029	0.2959	0.3163	0.2206	0.2465	0.2269	0.3060
29	199.02	0.2230	0.2470	0.2528	0.2772	0.2646	0.2192	0.2322	0.2841
30	97.03	0.2585	0.2078	0.3096	0.2242	0.2872	0.2094	0.2806	0.2229

The neighbor index gives the location of the nucleotide relative to the edited (or unedited) A, where negative values correspond to upstream nucleotides. The total number of *E*^1 ^sites here was 17,187, and about 378,000 *E*^0 ^sites (not all *E*^0 ^sites have all neighbors defined).

**Table 4:** Comparison of nucleotide distribution for sites in the vicinity of *E*^1 ^and *E*^0^ sites in interior loops.

Neighbor	χ^2^								
-30	97.88	0.2944	0.1880	0.2920	0.2255	0.2695	0.2264	0.2696	0.2345
-29	77.15	0.2535	0.2249	0.2540	0.2675	0.2320	0.2637	0.2525	0.2519
-28	91.61	0.1960	0.2207	0.2417	0.3417	0.2068	0.2516	0.2425	0.2992
-27	2.75	0.2247	0.2613	0.2596	0.2545	0.2246	0.2568	0.2670	0.2516
-26	4.52	0.2350	0.2720	0.2500	0.2430	0.2417	0.2750	0.2418	0.2415
-25	134.59	0.2420	0.2486	0.2239	0.2856	0.2580	0.2475	0.2576	0.2370
-24	133.46	0.2085	0.2218	0.3272	0.2425	0.2463	0.2384	0.2799	0.2353
-23	7.30	0.2390	0.2635	0.2617	0.2358	0.2432	0.2569	0.2544	0.2454
-22	52.35	0.2372	0.2504	0.2937	0.2187	0.2609	0.2629	0.2663	0.2099
-21	60.90	0.2339	0.2456	0.2587	0.2619	0.2468	0.2699	0.2291	0.2542
-20	59.59	0.2253	0.2372	0.2805	0.2570	0.2464	0.2351	0.2479	0.2706
-19	24.77	0.2506	0.2594	0.2494	0.2406	0.2527	0.2386	0.2653	0.2434
-18	58.31	0.2043	0.2669	0.2704	0.2584	0.2373	0.2470	0.2614	0.2543
-17	29.16	0.2112	0.2410	0.2724	0.2755	0.2197	0.2603	0.2561	0.2639
-16	135.38	0.2403	0.2471	0.3236	0.1890	0.2603	0.2502	0.2714	0.2180
-15	105.98	0.1849	0.2427	0.2664	0.3059	0.2228	0.2514	0.2560	0.2699
-14	54.33	0.2606	0.2152	0.3019	0.2222	0.2496	0.2447	0.2785	0.2272
-13	205.78	0.2890	0.1877	0.3317	0.1916	0.2795	0.2400	0.2769	0.2037
-12	228.23	0.1868	0.2349	0.2764	0.3019	0.2270	0.2715	0.2537	0.2478
-11	236.29	0.1555	0.2882	0.3067	0.2496	0.2183	0.2645	0.2662	0.2510
-10	89.14	0.2060	0.2653	0.2695	0.2593	0.2489	0.2497	0.2589	0.2425
-9	292.42	0.1939	0.3076	0.2877	0.2108	0.2594	0.2536	0.2579	0.2291
-8	30.94	0.2677	0.1964	0.3064	0.2295	0.2821	0.2113	0.2911	0.2155
-7	215.28	0.1802	0.2530	0.3269	0.2398	0.2382	0.2373	0.2774	0.2471
-6	101.36	0.2271	0.2597	0.3138	0.1995	0.2670	0.2347	0.2882	0.2100
-5	42.95	0.2734	0.2597	0.3004	0.1665	0.2709	0.2515	0.2845	0.1931
-4	233.23	0.2580	0.1873	0.2482	0.3066	0.2522	0.2413	0.2586	0.2480
-3	348.65	0.1739	0.3051	0.3311	0.1899	0.2357	0.2597	0.2793	0.2253
-2	801.12	0.1319	0.3376	0.2576	0.2729	0.2476	0.2556	0.2679	0.2289
-1	2126.16	0.2342	0.4720	0.0434	0.2504	0.2537	0.4022	0.2072	0.1368
1	1058.77	0.1864	0.2267	0.4276	0.1594	0.2681	0.2577	0.2739	0.2003
2	286.10	0.1901	0.3040	0.3206	0.1854	0.2432	0.2816	0.2590	0.2162
3	18.12	0.2495	0.2689	0.2165	0.2652	0.2667	0.2721	0.2063	0.2549
4	305.15	0.1705	0.2783	0.2719	0.2793	0.2413	0.2835	0.2240	0.2512
5	59.46	0.2358	0.2597	0.2849	0.2195	0.2703	0.2530	0.2620	0.2148
6	204.46	0.2500	0.2992	0.2671	0.1836	0.2644	0.2359	0.2846	0.2151
7	131.41	0.1930	0.3275	0.2314	0.2482	0.2255	0.2768	0.2469	0.2509
8	94.86	0.2310	0.2490	0.2996	0.2205	0.2724	0.2398	0.2667	0.2211
9	58.62	0.2569	0.2803	0.2656	0.1972	0.2750	0.2583	0.2473	0.2193
10	345.51	0.2084	0.3295	0.2893	0.1728	0.2616	0.2731	0.2471	0.2182
11	154.89	0.2131	0.2905	0.2166	0.2799	0.2549	0.3114	0.1850	0.2487
12	316.51	0.1599	0.3021	0.2146	0.3233	0.2358	0.2858	0.2061	0.2722
13	46.17	0.2451	0.2712	0.2496	0.2341	0.2759	0.2653	0.2314	0.2274
14	236.16	0.2052	0.2642	0.3278	0.2028	0.2637	0.2641	0.2672	0.2050
15	70.60	0.1946	0.2947	0.2711	0.2395	0.2284	0.2697	0.2589	0.2430
16	20.48	0.2917	0.2489	0.2549	0.2045	0.2835	0.2402	0.2521	0.2242
17	247.21	0.2104	0.2879	0.2879	0.2138	0.2648	0.2442	0.2498	0.2412
18	129.05	0.2228	0.2623	0.3005	0.2144	0.2697	0.2405	0.2672	0.2225
19	65.19	0.2967	0.2902	0.2103	0.2029	0.2839	0.2624	0.2254	0.2283
20	34.26	0.2335	0.2668	0.2505	0.2491	0.2532	0.2653	0.2278	0.2537
21	231.49	0.1794	0.2896	0.2905	0.2406	0.2380	0.2601	0.2474	0.2545
22	60.61	0.2726	0.2676	0.2488	0.2110	0.2783	0.2413	0.2402	0.2402
23	65.14	0.2495	0.3083	0.2519	0.1903	0.2684	0.2726	0.2515	0.2074
24	37.99	0.3123	0.2029	0.2840	0.2008	0.3094	0.2200	0.2591	0.2115
25	110.08	0.2531	0.2422	0.3048	0.1999	0.2977	0.2215	0.2736	0.2072
26	215.24	0.2745	0.2894	0.2753	0.1608	0.2691	0.2531	0.2552	0.2226
27	17.11	0.2703	0.2640	0.2459	0.2198	0.2760	0.2536	0.2357	0.2347
28	51.46	0.2334	0.2456	0.2681	0.2528	0.2520	0.2525	0.2367	0.2588
29	89.77	0.2545	0.2778	0.2366	0.2311	0.2690	0.2449	0.2223	0.2637
30	152.90	0.2346	0.2363	0.3191	0.2100	0.2785	0.2206	0.2728	0.2281

The neighbor index gives the location of the nucleotide relative to the edited (or unedited) A, where negative values correspond to upstream nucleotides. The total number of *E*^1 ^sites here was 9,711, and about 97,000 *E*^0 ^sites (not all *E*^0 ^sites have all neighbors defined).

**Table 5:** Nucleotide enrichment for several locations neighboring an editing sites

neighbor	helix	interior	**pattern reported in **[41]
up18:A	0.9468	0.8720	C = T > G
up18:C	1.0250	1.0727	
up18:G	0.9590	1.0313	
up18:T	1.0635	1.0149	
			
up9:A	0.9579	0.7649	G > A = T
up9:C	1.1032	1.1904	
up9:G	1.0449	1.1040	
up9:T	0.8916	0.9270	
			
up1:A	1.1147	0.9294	T = A > G
up1:C	0.9981	1.1554	
up1:G	0.3239	0.2256	
up1:T	2.2055	1.7022	
			
dn7:A	0.8456	0.8678	T > C > G = A
dn7:C	1.2455	1.1647	
dn7:G	0.9262	0.9433	
dn7:T	0.9932	0.9908	
			
dn9:A	1.0000	0.9407	U > A > G
dn9:C	0.9352	1.0777	
dn9:G	0.9574	1.0675	
dn9:T	1.1277	0.9083	
			
dn10:A	0.8874	0.8125	A = T > G = C
dn10:C	1.1173	1.1853	
dn10:G	1.1126	1.1540	
dn10:T	0.9114	0.8079	
			
dn13:A	0.8481	0.8988	T = A > G
dn13:C	0.9946	1.0219	
dn13:G	1.1912	1.0726	
dn13:T	1.0215	1.0281	
			
dn15:A	0.7704	0.8653	G > T > A = C
dn15:C	1.1095	1.0859	
dn15:G	1.1407	1.0447	
dn15:T	0.9629	0.9884	

Nucleotide distributions at certain locations around editing sites, reported to exhibit nucleotide biases [41]. For each of the sites, we present the probability to have a given nucleotide N when the 0 location adenosine is edited, divided by the probability of that nucleotide regardless of whether the 0 adenosine is edited: *P*(N|0 adenosine is edited)/*P*(N). Values > 1 indicate enrichment of N for edited sites, and < 1 indicate depletion. upX = X nucleotides upstream of site 0, dnX = X nucleotides downstream. The probabilities are given for editing sites in helices and interior loops separately, but are very similar for both. For comparison, we present the patterns reported in [41].

### Thermodynamic Calculations

Finally, we study the effect of thermodynamic stability on editing efficiency. For each genomic neighborhood, we look at the thermodynamic average over all the low free-energy structures. The laws of statistical mechanics give us the probability that the RNA is in a specific secondary structure *n*,(1)

where *T *is the temperature in degrees Kelvin, *k_B _*is Boltzmann's constant and *Z *is defined by the sum(2)

where the label *n *runs over all available foldings, and *G_n _*are the respective free energies. In practice, we only use those folds generated by MFOLD which are expected to be all folds relevant at human-body temperature. We may now, for example, calculate the probability of some particular site to be in a helix,(3)

where  is the indical function, defined by(4)

Similarly, one may calculate the probabilities for all other substructures.

**Figure 10:**
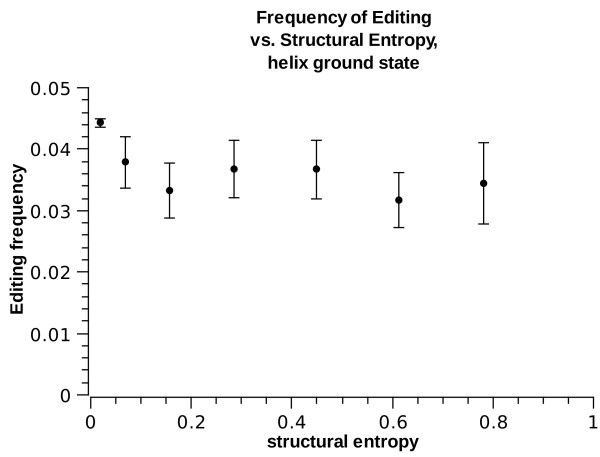
***E*^1 ^frequency vs. structural entropy for ground state helix**.

**Figure 11:**
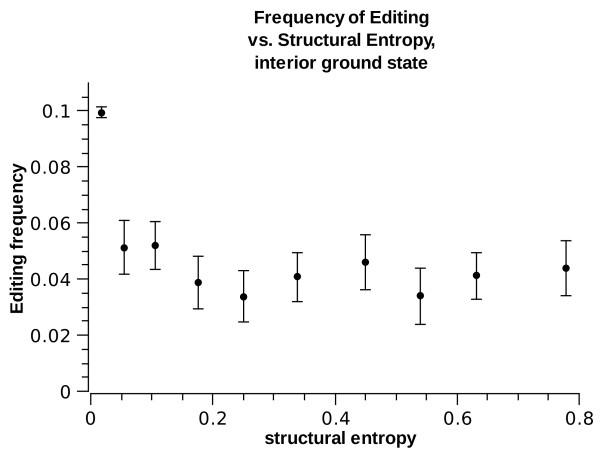
***E*^1 ^frequency vs. structural entropy for ground state interior-loop**.

Let *S *denote the set of possible substructures,

We define a site's *structural entropy *to be

where  is the frequency of site *i *being in substructure of type *s*. This entropy is a measure of the thermodynamic volatility of the site's substructure: if a site is always in the same substructure (e.g. the site is always in a helix), it will have zero structural entropy. If, however, the site's substructure fluctuates, for example between a helical structure and a loop structure, it will have higher structural entropy. The structural entropy of a site with equal probability to be in two difference substructures is ln(2) = 0.7. The highest possible structural entropy is of a site which spends equal time in each of the possible substructures. Figs. 10 and 11 show the frequency of *E*^1 ^as a function of the structural entropy, for sites whose lowest free-energy structure is helix or interior-loop separately. Interestingly, A-to-I editing is enriched for sites with low structural entropy, i.e. having a well-defined low energy micro-structure. A wobbling state, fluctuating between two or more possible structures is less well edited. This holds regardless of the ground-state structure, but the effect is stronger for interior loops: sites with a well-defined interior loops structure are twice more likely to be edited compared with sites whose ground state structure is also an interior loop but having even 1% probability to be in other structures.

Analysis of a large dataset of secondary structures of putatively edited *Alu *repeats reveals that structural motifs are indeed important in determining the A-to-I editing efficiency of a given site. Most notably, we highlight the strong preference for editing of adenosines within short symmetrical internal loops. Moreover, the microstructure also has modest but noticeable effect on the cis-preferences of the ADARs. Long perfect dsRNA duplexes are often considered to be the best target for editing by ADARs. Here we find that sites adjacent to the edge of helices (cePos = 0) are even more efficiently edited. Averaged over our whole database, adenosines deep within (cePos > 10) long (> 30 bp) perfect helices are indeed edited more efficiently than the average adenosine in a helix - we find 1625 such sites, with editing frequency 8.2%, compared to only 4.4% for the average helix site. However, this is still lower than the average frequency for interior loops, 9.1%. Moreover, focusing on single A-C mismatches within a helix (i.e. cePos = 0 sites having neighboring helices on both sides and C as the opposite nucleotide) raises the frequency to 19%. Finally, choosing also the optimal neighbors, i.e. T upstream and G downstream, raises the editing frequency as observed in our database to 37% ! We stress again that these frequencies should not be regarded as the true editing efficiency, but rather as a relative measure. Yet, one is able to conclude that the best way to engineer an efficient editing site is not to put it in a long perfect duplex, but rather to be in a single mismatch within a duplex.

Interestingly, the 100% edited E1 site in the NARF gene [25], fits nicely with these engineering rules - it is a cePos = 0 site in a symmetric loop, with C opposite to it and T and G in the upstream and downstream sites, respectively. However, the strand length there is 3 and not the optimal 1. An editing site that fully adheres to the above "rules" is the amber/W one of the hepatitis delta virus antigenome (genotype I) [46]. This site is critical for the virus to assemble viral particles and to be infectious [47]. Given the high adaptivity of viruses, it is not surprising to find that this site fits with all of the above: it is located in a single A-C mismatch within a helix (cePos = 0 and loop length = 2), and has T and G as its immediate neighbors.

However, the Q/R site in GluRB does not fit to our observations. It lies within a rather long (17 bp) helix, with cePos = 5, with C (rather than the optimal T) upstream and G downstream [48]. Yet, this site is also 100% edited. Apparently, there is still much more to learn about the characteristics of editing by ADARs, beyond the information presented in the present study. One possible explanation is that this site in known to be edited only by ADAR2 [49]. The two editing enzymes ADAR1 and ADAR2 are known to have overlapping, but distinct, preferences [36-38,50,51]. However, our approach does not allow us to distinguish between them. It was recently shown that editing of mouse B1 and B2 SINEs is mediated by both enzymes [39]. Some sites within these repeats are ADAR1 specific, some are ADAR2 specific and some are edited by both. It is not yet clear which enzyme is the main one in terms of *Alu *editing in human. Since our database is based on mRNA sequences from a wide range of tissues, it is possible that it characterized mainly the profile of the widely-expressed ADAR1 rather than that of ADAR2 which is expressed mainly in the brain. It is thus likely that some of the preferences identified in this work characterize ADAR1 and are therefore not present in the GluRB ADAR2-specific site. The discrepancies between nucleotide distributions around the editing sites reported above and those reported by [41] for ADAR2 sites might also attest for differences between the ADAR2 profile and the one characterizing our dataset, probably a mix of the two enzymes, with larger weight of ADAR1.

In an attempt to estimate the differences between the two enzymes, we compared 4657 editing sites supported by 13805 brain mRNAs, where both ADAR1 and ADAR2 are present, and 1684 sites residing in 10186 non-brain mRNAs, presumably edited mostly by ADAR1 (tissue-origin was determined by UCSC annotation [52]). The patterns observed were similar but not identical. For example, 1376 of the 2966 brain sites residing in a helix (46.4%) had a G in the dn1 position, compared to 452 out of 1076 (42.0%) in non-brain sites in a helix (*p*-value 0.013). However, differences were not statistically significant upon Bonferroni-correcting for multiple testing. Thus, a larger and better dataset (fully characterized in terms of of the tissues studied) is required in order to study the small tissue differences between the preferences of the two ADAR enzymes.

## Conclusions

Using a dataset of 29,971 editing sites within Alu repeats, we analyzed the editing preferences. We found that the micro-structure a site resides in affects its editing frequency. In addition, the sequence motifs characterizing editing sites vary with the micro-structure. We have also shown that structural entropy and thermodynamic stability play a role in determining editing efficiency. We find that the probability of a nucleotide fluctuating between a number of possible structures to be edited is lower than the weighted average of the probabilities for each possible structure alone. This provides a hint as to the rate of the A-to-I editing process compared to the relaxation time scales controlling the transition between the possible folds.

Taken together, the results presented here could be of help in revealing the complex nature of the ADARs editing profile.

## Methods

We construct a list of putative editing sites within Alu repeats following the method presented in [23,24]. That is, we use mismatches in the relatively clean RNA sequences, rather than the much larger but noisier EST data. We use UCSC alignments of human RNA sequences to the genomes http://genome.ucsc.edu[52] and record all mismatches in these alignments. Then, known SNPs are removed, and the list is intersected with Alu locations, to obtain a set of mismatches within Alu repeats. A-to-I editing sites in Alu repeats tend to occur in clusters, we thus take only clusters of three or more consecutive identical mismatches. While this process is not inherently biased towards any specific type of mismatch, 98% of the mismatches found are A-to-G, suggesting that although these sites are typically supported by a single mismatch, they do represent true A-to-I editing sites with a low level of false-positives.

We then construct the predicted secondary structures using the following procedure: (a) for each site in our list, its *Alu *was located in the genome. Then, the nearest antisense *Alu *was located, and the genomic neighborhood that includes all nucleotides in and between the two *Alu*s was taken, along with 200 extra nucleotides on each side. 61% of the inter-*Alu *distances are less than 1000 nucleotides, 22% more lie between 1000 and 2000, 9% are between 2000 and 3000 and the remaining 8% strech from 3000 to 6800 nucleotides.

(b) Neighborhoods having > 400 bp overlap on the same strand were merged into a single neighborhood encompassing both. This step resulted in 3,276 neighborhoods, containing 29,971 putative editing sites. RNA segments corresponding to these neighborhoods were folded using MFOLD, resulting in predicted secondary structures.

The accuracy of RNA secondary structure prediction by current dynamic programming algorithms (such as the MFOLD software) is moderate, up to 73% [53]. Yet, while false structure predictions would inevitably introduce noise to our analyzed data, the large sample size should allow for detecting a signal. Moreover, one should bear in mind that the RNA structures we consider - long and almost perfect dsRNAs - are relatively easy to analyze, and thus the accuracy of the folding algorithms is expected to be much higher than the above quoted rate.

(c) We parsed the output of MFOLD into a relational database containing all the information about the secondary structures in which the various sites reside (the basic substructures are given in fig. 1).

(d) All adenosines in the genomic neighborhoods' sites were classified: We find 29,971 putative editing sites within Alu repeats, denoted by *E*^1 ^and 590,206 adenosines within *Alu *repeats that were not detected as editing sites, denoted by *E*^0^. The adenosines which are not in *Alu *repeats do not enter our analyses. In the following, we use the *E*^0 ^sites as a control set. It should be stressed that the sensitivity of the bioinformatic algorithms for detecting editing sites is rather low, mainly due to the low coverage, low editing efficiency of most sites and tissue origin of the available sequences. For example, the observed editing efficiency averaged over all *Alu*s, which is 0.048, is probably lower than the actual value. Therefore, the set of *E*^0 ^sites should not be thought of as sites that are never edited, but rather as a background, maybe slightly depleted in editing sites. On the other hand, the set of *E*^1 ^contains, with high precision, only edited sites [23].

## Authors' contributions

YK carried out the RNA folding computations, prepared the foldings database and performed the statistical analysis. EE designed the study and prepared the editing sites database. Both authors read and approved the final manuscript.
